# Periodicity of varicella-zoster virus in the presence of immune boosting and clinical reinfection with varicella

**DOI:** 10.1186/s12976-015-0002-5

**Published:** 2015-04-11

**Authors:** Igor A Korostil, James G Wood, David G Regan

**Affiliations:** The Kirby Institute, University of New South Wales, Sydney, 2052 Australia; School of Public Health and Community Medicine, University of New South Wales, Sydney, 2052 Australia

**Keywords:** Zoster, Shingles, Periodicity, Varicella, Recurrence

## Abstract

**Background:**

Clinical reinfection with varicella is normally ignored in mathematical transmission models as it is considered too rare to be important.

**Methods:**

We apply basic bifurcation analysis to a simple mathematical model of varicella-zoster virus (VZV) transmission incorporating reinfection.

**Results:**

We demonstrate that under certain conditions this model can exhibit periodic behaviour as opposed to what is observed in VZV models that ignore the possibility of repeat varicella attacks. Periodicity can be induced by a combination of immune boosting and reinfection while the impact of zoster (shingles) recurrence on the onset of periodicity is negligible.

**Conclusions:**

Our results suggest that mathematical models of VZV may benefit from inclusion of repeat varicella.

**Electronic supplementary material:**

The online version of this article (doi:10.1186/s12976-015-0002-5) contains supplementary material, which is available to authorized users.

## Background

Varicella-zoster virus (VZV) is first manifested as varicella (also known as chickenpox), a rash that turns into open lesions. In the absence of vaccination, this usually happens when a person is young. While the symptoms clear within a week or so, VZV remains latent in the nervous system and may reactivate to cause a rash called herpes zoster (HZ) or shingles. Why this reactivation happens remains unclear, although it is generally assumed that onset is related to reduced levels of cellular immunity [1-3].

Clinical reinfection with varicella is thought to be rare and is typically not accounted for in epidemiological models of VZV transmission [4]. However, observational studies suggest that this may be inaccurate, with a number of literature reports suggesting that reinfection occurs with moderate frequency. In particular, two US studies [5,6] based on active surveillance data indicate that a substantial proportion of varicella cases (between 4.5% and 25%) report a prior history of varicella infection. A smaller study demonstrated laboratory evidence of prior infection in 8 out of 115 (7%) patients with clinical varicella infection [7]. There are also case reports of multiple varicella attacks in healthy children [8]. Hence, it appears that prior infection does not always result in sustained protective immunity against subsequent varicella reinfection and there is a case for considering this process more closely in models.

Repeat HZ attacks in immunocompetent individuals have also typically been excluded in epidemiological modelling studies [4,9,10], under the assumption that repeat attacks primarily occur in immunocompromised individuals. However, second attacks were observed by Hope-Simpson [1] with 8 out of 192 cases recorded in his series being a repeat event. Repeat cases were also observed by Wilson in a rural UK practice with 6 out of 151 cases identified as repeat events [11]. More recently, a US study found that 95 out of 1,669 individuals with a medically documented episode of HZ had two or more attacks (8 individuals had three or more attacks) [12]. While immunocompromised patients had a higher incidence of recurrence, the rate of recurrence in immunocompetent patients was similar to the rate of first attacks reported in other studies.

The Hope-Simpson hypothesis that the immune response of someone who has been exposed to varicella can be strengthened (boosted) via subsequent exposure to VZV [1] has been supported by both clinical and modelling studies [3,13] although there are studies which failed to observe evidence of boosting [14]. The degree of exposure that would be required to provide an immune boost remains unknown. The VZV modelling studies of which we are aware implement boosting by assuming that individuals with weakened immunity are boosted to full immunity at a fraction of the rate of at which they would be becoming infected with varicella (this rate is often referred to as a force of infection) [4,10,15-17]. In a recent work on the transmission of pertussis, the potential for the rate of boosting to exceed the force of infection was explored and shown to induce both cyclical behaviour and the potential for disease recurrence following immunisation [18]. It was suggested that immune boost could occur even if the exposure dose is too small to be likely to cause clinical infection. This is supported by the observation that immune memory cells respond more rapidly and to lower doses of antigen when primed through previous exposure than in naive individuals [19,20].

In this paper, we consider a simple mathematical model of varicella-zoster virus transmission incorporating clinical reinfection with varicella, immune boosting requiring either more or less exposure to VZV than primary infection, and HZ recurrence. Our aim is to investigate whether the behaviour of this model qualitatively changes when its parameters are varied within ranges selected based on current knowledge of VZV. Specifically, we are interested in identifying conditions whereby there is switching from stable equilibrium behaviour (that modelling studies normally focus on) to periodic behaviour. While varicella is known to exhibit periodicity [3,21], the evidence for HZ periodicity is still unclear, with studies both supporting periodically-varying incidence [22,23] and failing to find evidence of it [24,25]. The majority of epidemiological modelling studies have not explicitly incorporated periodic VZV transmission (e.g. transmission rates modelled as sinusoid) or observed VZV-related periodicity, although extensive investigations of this periodicity have been conducted in the context of chaotic dynamics (see e.g. [26]).

## Methods

**Figure 1:**
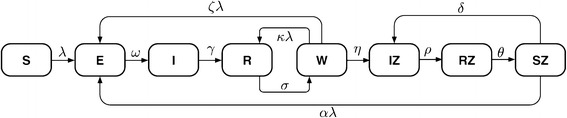
Schematic diagram of a simple VZV model. The included compartments contain the susceptible to varicella (S), individuals exposed to varicella (E), those who are infected with varicella (I), who have recovered and are protected from VZV (R), who are weakly immune (W), i.e. individuals partly susceptible to varicella and HZ, individuals infected with zoster (IZ) and those who have recovered from zoster and are immune to VZV (RZ) and individuals who lost immunity after recovery from zoster and are susceptible to VZV (SZ). The arrows indicate movements between compartments at the rates marked by Greek letters. The rates are explained in Table 1.

We consider a compartmental model as illustrated schematically in Figure 1. This model describes VZV transmission in a large population (i.e. transmission characteristics are averaged over large groups of individuals) and is adapted from the basic structure of existing varicella models but with some additional components. The model is of SEIR-type, with these compartments corresponding to susceptible, exposed, infected or immune to varicella infection individuals, respectively. The model also includes additional states representing reduced immunity and susceptibility to zoster (W), infection with zoster (IZ), recovery from zoster and immunity (RZ) and, finally, susceptibility to varicella and zoster following the loss of immunity acquired after recovery from zoster (SZ). This structure is essentially identical to the basic set of states in the absence of vaccination in the model of Brisson et al. [4], which has been used as the basis for a number of subsequent studies [10,16,17]. However, we also introduce 4 additional features, which we describe in turn here. First, we allow for recurrence of HZ, with residence in the state RZ set to be temporary (it is permanent in the papers derived from [4]) with an exit rate parameter *θ*. Second, we allow for individuals in the reduced immunity state W to be boosted at a rate that is higher than the force of infection (i.e. the boosting weight parameter *κ* is allowed to be >1). This is based on the observation that individuals previously exposed to infection make immune responses to lower infectious doses than naive individuals and is included to test whether this can produce periodicity in varicella incidence. Finally, we make two further changes that deal with repeated varicella infections. The first of these allows for recurrence of varicella from the reduced immunity state W, a transition that is supported by the evidence of second varicella attacks described in the introduction (proportional to the varicella force of infection *λ* with relative hazard *ζ*). The final change also allows for new primary infection with varicella from the SZ state (with relative hazard *α*) which does not appear implausible given rates of zoster recurrence. The latter suggest that loss of immunity may be sufficient to experience a second primary VZV infection (although we are not aware of any published data that would clearly support possibility of the SZ to E transition).

**Table 1:** **Model parameters**

**Parameter**	**Notation**	**Base value, refs.**	**Range**
Varicella transmission coefficient	*β*	325 [27]	120–500
Rate of becoming infectious with varicella	*ω*	26 [21]	20–32
*the base value corresponds to the average latent period of ≈ 14 days*			
Varicella recovery rate	*γ*	50 [21]	40–60
*the base value corresponds to the average infectious period of ≈ 7.3 days*			
Loss of full immunity rate	*σ*	0.10 [4,9,17]	0.0125–4.0
*the base value corresponds to the average duration of “full” immunity of 10 years*			
Varicella force of infection reduction coefficient for those	*ζ*	0.10 [assumed]	0.0–1.0
who become reinfected following clearance of varicella			
Varicella force of infection reduction coefficient for those	*α*	0.90 [assumed]	0.0–1.0
who become reinfected following HZ attack			
Boosting coefficient	*κ*	3.0 [assumed]	0.0–20.0
Birth/death rate	*μ*	0.0122 [29]	0.01–0.05
*the base value corresponds to the average life expectancy of ≈ 82 years*			
HZ reactivation rate	*η*	0.05 [4,15,17]	0.0001–2.0
HZ recovery rate	*ρ*	52 [17]	46–58
*the base value corresponds to the average infectious period of ≈ 7 days*			
Rate of HZ reactivation after loss of immunity following recovery from HZ	*δ*	0.01 [12,23]	0.00–0.10
Rate of loss of immunity to VZV after recovery from HZ	*θ*	4.0 [assumed]	0.00–12.0
*the base value corresponds to immunity lasting 3 months on average*			
Relative VZV infectiousness of HZ	*p*	0.001 [10,17]	0.00–0.10

All rates in this table are per year. Note that *β* and *p* are used to define the force of infection (2).

As we aim to make our model reasonably parsimonious to facilitate understanding of its equilibrium behaviour in the presence of the above-mentioned new features, we assume a very simple demography with a constant death rate *μ* in all states, which is balanced by births so that the population size is conserved. This clearly makes our model less realistic but also reduces the number of model parameters by at least a dozen as compared with age-stratified models (see, for example, [4]). Hence, the model is described by the following system of nonlinear ordinary differential equations:

(1a) $$\begin{array}{*{20}l} \dot{\mathbf{S}}&=-(\lambda+\mu)\mathbf{S} +\mu \end{array} $$

(1b) $$\begin{array}{*{20}l} \dot{\mathbf{E}}&=-(\omega+\mu)\mathbf{E}+\lambda \mathbf{S}+\zeta\lambda \mathbf{W}+\alpha\lambda\mathbf{SZ} \end{array} $$

(1c) $$\begin{array}{*{20}l} \dot{\mathbf{I}}&=-(\gamma+\mu) \mathbf{I}+\omega \mathbf{E} \end{array} $$

(1d) $$\begin{array}{*{20}l} \dot{\mathbf{R}}&=-(\sigma+\mu) \mathbf{R}+\kappa\lambda \mathbf{W}+\gamma \mathbf{I} \end{array} $$

(1e) $$\begin{array}{*{20}l} \dot{\mathbf{W}}&=-((\kappa+\zeta)\lambda+\eta+\mu)\mathbf{W}+\sigma \mathbf{R} \end{array} $$

(1f) $$\begin{array}{*{20}l} \dot{\mathbf{IZ}}&=-(\rho+\mu)\mathbf{IZ}+\eta \mathbf{W}+\delta \mathbf{SZ} \end{array} $$

(1g) $$\begin{array}{*{20}l} \dot{\mathbf{RZ}}&=-(\theta+\mu)\mathbf{RZ}+\rho\mathbf{IZ} \end{array} $$

(1h) $$\begin{array}{*{20}l} \dot{\mathbf{SZ}}&=-(\delta+\alpha\lambda+\mu)\mathbf{SZ}+\theta\mathbf{RZ} \end{array} $$

where it is assumed that

$$\mathbf{S}+\mathbf{E}+\mathbf{I}+\mathbf{R}+\mathbf{W}+\mathbf{IZ}+\mathbf{RZ}+\mathbf{SZ}=1, $$

and the varicella force of infection is defined as

(2) $$  \lambda=\beta (\mathbf{I}+p \mathbf{IZ}).  $$

The system coefficients are described and supporting references supplied in Table 1. The base values should be understood as some values from the specified ranges (i.e. plausible values) convenient as the starting point for bifurcation analysis. Concretely, we would like to be able to vary any parameter value keeping other parameters at their base values to obtain an initial Hopf point. The base value for varicella transmission coefficient *β* has been specified based on the reported average basic reproductive ratio (*R*_0_=6.5) for 11 European countries [27]. It can be easily verified (using, for example, the next generation approach described in [28]) that for system (1)

$$R_{0}=\frac{\omega\beta}{(\omega+\mu)(\gamma+\mu)}. $$

Hence, using the base values for *ω*,*μ* and *γ* provided in Table 1, we see that *β*≈325 year^−1^. The rate of loss of full immunity *σ* is unknown but attempts to estimate it have been made previously, hence we cite three relevant modelling studies. We set a base value for *σ* corresponding to the average duration of full immunity of 10 years and let *σ* take a range of possible values from 0.0125 (“full” immunity for 80 years) to 4.0 (“full” immunity for only 3 months). The boosting coefficient *κ* can be interpreted as a measure of how much exposure is required for immune boosting as compared with that required for primary infection. We allow it to vary from 0 to 20. The birth/death rate *μ* has been set to correspond to the current Australian life expectancy at birth [29]. There are no readily available data to directly inform parameter *p*, which characterises the contribution of HZ to the varicella force of infection. Some VZV modelling studies have assumed that this contribution is so small that it can be ignored [30] while others attempted to estimate it. In particular, *p*=5.4×10^−7^ was estimated in [4] and [16], while *p*=3.11×10^−6^ was estimated in [31]. A substantially larger value of 0.05 was used in [17]. Hence, we allow *p* to vary between 0 and 0.1. The rate of HZ reactivation after loss of immunity following recovery from HZ *δ* is allowed to take values between 0 (no reactivation) and 0.1. Loss of immunity to VZV after recovery from HZ is characterised by parameter *θ* which ranges from 0 (no loss of immunity) to 12.0 (immunity lost after only 1 month). Finally, parameters *ζ* and *α* have been introduced to allow for reinfection with varicella. In the base case we assume that *α*=0.90 (i.e. the force of varicella infection on those who have recovered from HZ is reduced by only 10% as compared to that on someone susceptible to primary varicella infection) and allow *α* to take values from 0 (those who have had HZ cannot be reinfected with varicella) to 1. The base value for *α* is high not due to epidemiological considerations but because we determined that for substantially lower values of *α* system (1) does not deviate from stable equilibrium behaviour no matter what values the other parameters take. This is evident from the results presented in the next section. We set the base case value for *ζ* at 0.1, which means that the force of varicella infection on those who have recovered from varicella and have not had HZ is reduced by 90% as compared to that on individuals susceptible to primary infection.

In order to detect the periodic dynamics we systematically searched for Hopf points using the XPPAUT [32] bifurcation analysis software. A Hopf point is a point where a bifurcation from a branch of stable equilibrium to a branch of periodic oscillation occurs [33,34]. This point can be detected by varying a single model parameter while the other parameters are kept fixed. Having found the Hopf point, we can investigate where it would be if another parameter were given a different value. In this way we obtain a set of Hopf points which form a so-called Hopf curve.

## Results

**Figure 2:**
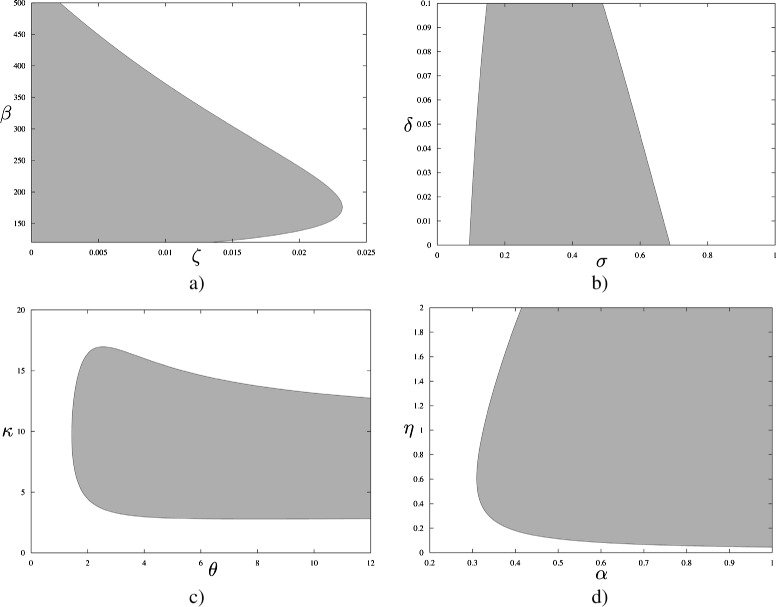
Hopf curves for selected parameter pairs: **(a)** *β* is a varicella transmission coefficient and *ζ* is a varicella force of infection reduction coefficient for those who become reinfected following clearance of varicella; **(b)** *σ* is a loss of full immunity rate and *δ* is a rate of HZ reactivation after loss of immunity following recovery from HZ; **(c)** *θ* is a rate of loss of immunity to VZV after recovery from HZ and *κ* is a boosting coefficient; **(d)** *α* is a varicella force of infection reduction coefficient for those who become reinfected following HZ attack and *η* is a HZ reactivation rate. Shaded areas limited by these curves correspond to periodic solutions of (1).

**Figure 3:**
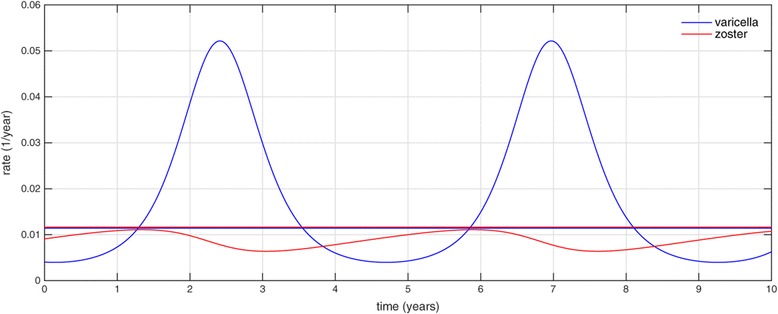
Varicella and zoster incidences produced by (1) reach a steady equilibrium (i.e. they are both straight lines) if *ζ*=0.05,*κ*=0.6,*α*=0.01 and other parameter values are as in Table 1; if instead we take *ζ*=0.01,*κ*=3.0,*α*=0.95, varicella and zoster incidences exhibit a steady periodic behaviour.

We constructed the Hopf curves for all pairs of model parameters. Four examples of these curves are shown in Figure 2 while the complete set of curves (78 figures in total) can be found in Additional file 1. The shaded areas in each plot represent regions of each two-parameter space in which solutions are periodic (with other parameters fixed at base-case values as in Table 1), while the complementary area corresponds to solutions converging to a (globally) stable equilibrium. Example of solutions corresponding to either area are shown in Figure 3.

We found that for certain parameters it was easy to obtain periodic behaviour for most or all of their values taken from within the ranges given in Table 1. Specifically, for all values of *β* under 400 we could normally observe oscillations, and this was also the case for *β* under 500 when *η* and/or *σ* were increased or *μ* and/or *ζ* were decreased as compared to their base values. This is illustrated in Figure 2a, where one can pick any value of *β* and for that value there is always a range of values of *ζ* to select to ensure periodic behaviour. For the rate of becoming infectious with varicella, *ω*, oscillations were possible for all values except when the HZ recovery rate *ρ* was at the bottom of its range (around 46-48) and *δ* was approaching 0.017. Similarly, the entire range for the varicella recovery rate *γ* values allowed for oscillatory solutions except when *δ* and *θ* were varied. The HZ recovery rate, *ρ*, behaved in the same way. Note that *β*,*ω*,*γ* and *ρ* are considered well informed.

Another group of parameters turned out to be unnecessary to support oscillatory behaviour. One of these parameters was *ζ*: it usually had to be under 0.02, i.e. the force of varicella infection on those who cleared varicella previously had to be reduced by at least 98%. This means that *ζ* could as well be zero, as shown in Figure 2a. Parameter *δ* (the rate of HZ reactivation following recovery from HZ) tended to be under 0.07 unless *κ*,*μ*,*ζ* and *σ* were manipulated. As shown in Figure 2b, *δ* can be as large as 0.1 if *σ* is somewhere between 0.2 and 0.55, but it can also be zero. Similarly, *p* was consistent with oscillations when small (of the order 10^−3^) or equal to zero in line with the values estimated in [4,16,31] but much less than *p*=0.05 used in [17].

At last, there were six parameters that allowed for oscillatory behaviour when the ranges of values they could vary in were narrowed to some extent. Specifically, the boosting parameter *κ* had to exceed 2.5. Figure 2c illustrates that in the grey area where the values of *κ* and *θ* corresponding to oscillations are, *κ* is clearly much larger than 1. Parameter *μ* was restricted to under 0.015 (the corresponding life expectancy of at least 66.7 years) unless *η* increased and then *μ* could even be 0.04. The loss of full immunity rate, *σ*, had to exceed 0.1 (duration of immunity under 10 years) but was usually limited by 0.8 (or 1.1 if *κ* was approaching 8). The values of *θ* under about 2 or a little less were not compatible with oscillations (see Figure 2c) and neither was the HZ reactivation rate *η* under 0.05, which is evident from Figure 2d. At last, *α* had to be over 0.7 in most cases - a notable exception is shown in Figure 2d where *α* can approach 0.3 assuming that *η* is around 0.5.

**Figure 4:**
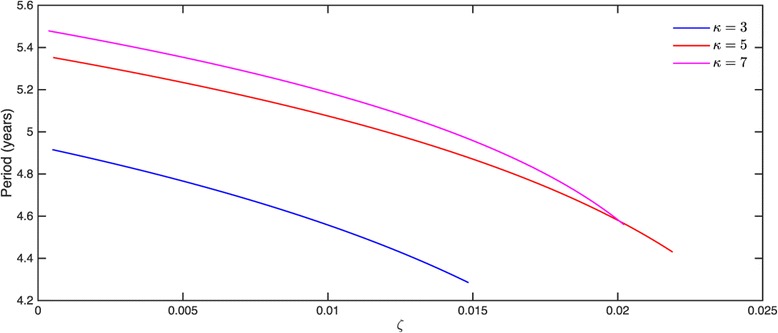
Changes in the period of steady oscillations for selected values of *κ*.

The period of oscillation itself tended to vary between 4 and 5 years for most parameter combinations that are consistent with periodic solutions, which is illustrated in Figure 4. We were unable to obtain solutions with a period close to 1 year using parameter values from the ranges given in Table 1. This period would be of interest because varicella incidence has been reported to exhibit yearly peaks (see, for example, [23]).

It is pertinent to note that periodic solutions typically corresponded to repeat varicella incidence that we would call substantial. For example, varying *ζ* while all other model parameters are fixed results in detection of a Hopf point at *ζ*=0.01314. This point corresponds to the yearly incidence of repeat varicella periodically reaching a maximum of 1.05% of the modelled population. The yearly HZ incidence is also periodic in this circumstance averaging 0.00215% and peaking at around ±0.0023%.

## Discussion

In this study, we developed a simple VZV transmission model with novel features to explore the influence of boosting and disease recurrence on the potential for periodicity in varicella and zoster disease incidence. We relied upon bifurcation analysis of this model to assess the relationship between key parameters and the existence of periodic solutions.

In comparison with other published VZV transmission models, our model has four additional features: repeated HZ attacks and rates of boosting exceeding the force of infection are allowed, while we also include the possibility of clinical reinfection with varicella from a waned immunity state and in individuals who have experienced zoster.

We verified that our model can produce oscillations in both varicella and zoster which is similar to the situation described in an extensive Japanese study covering a total of 48,388 patients with zoster [23]. However, some of the model features were shown to be redundant in the context of periodicity (i.e. periodic solutions could still be obtained if these features were removed from the model):

- reinfection with varicella for individuals who recovered from varicella and are in the weakly immune state W (as mentioned in Results, *ζ* can be zero);
- reactivation of HZ following recovery from zoster (*δ*=0 is possible);
- contribution of HZ to varicella force of infection (*p* can be zero).

On the other hand, the following requirements turned out essential to produce stable periodic solutions:

- duration of full immunity to VZV is no longer than 10 years (*σ*>0.1);
- duration of immunity to VZV following recovery from HZ should not exceed 6 months (*θ*>2)
- immune boosting requires less exposure than primary varicella infection (*κ*≥2.5);
- unless the average HZ reactivation rate *η* is only slightly under 0.2 (that is 4 times its base value), those who have had HZ and are in contact with individuals infected with varicella have little or no added protection against varicella as compared with individuals never infected with VZV (*α*>0.7).

It is not immediately clear how to assess plausibility of these requirements. The existing data can only give some indications regarding the average time needed to return from I to I or progress from I to IZ. Since the immunity in question is the full immunity corresponding to the average time needed to progress from *I* to *W* and the latter is included to implement the Hope-Simpson hypothesis (i.e. the very existence of this state is not an established fact), we should note that there is no data to suggest that *σ*>0.1 may be unrealistic. There is also not enough data to clarify the average duration of staying in the RZ compartment.

The exposure required for boosting VZV immunity is not well understood but the Hope-Simpson hypothesis is widely applied in modelling studies with *κ*≤1. Nonetheless, we are not aware of any strong evidence in support of this restriction which precludes *κ*≥2.5 (a relevant discussion on why *κ*>1 is likely to be valid for pertussis can be found in [18]).

Based on the Hope-Simpson hypothesis, our model makes individuals recovered from HZ immune but we do not know how long this immunity should last on average. Our analysis shows that we can obtain periodicity if this duration is under 6 months which we can not consider implausible until there is evidence to prove otherwise. One would also expect that being in the post-zoster state individuals may be able to strengthen their immunity when exposed to varicella and our results do not contradict this expectation. However, they indicate that the onset of oscillatory behaviour would occur if this protection, if gained at all, were insignificant: *α*>0.7 means that someone who have had HZ, then was immune for a while and then became susceptible to VZV is becoming reinfected with varicella at an average rate reduced by up to 30% at best or much less, as compared with a rate this individual would be becoming infected with varicella at if he or she was exposed to varicella for the first time. As those who have had HZ are most often elderly and their immunity is weakened by age, there is a reason to believe that they may become susceptible to varicella reinfection via contact with infectious varicella cases and the immune boosting mechanism may not work for these individuals very well. The absence of a strong evidence base for such cases does not necessarily preclude them being common. For example, routine reporting schemes are frequently recording numerous “unspecified VZV” diagnoses [35] which could be either HZ or varicella. Separating varicella and HZ diagnoses can be difficult. Laboratory testing is infrequent, and the ability to differentiate re-infection from reactivation is therefore based on the disease syndrome rather than confirmation through viral testing. As discussed by Volpi et al. [36] the clinical diagnosis of HZ is subject to error, with the Shingles Prevention Study reporting that 24% of clinical HZ diagnoses were not confirmed to have HZ by laboratory testing [13] and a UK study found 17% (48 out of 278) of GP clinical HZ diagnoses to be incorrect [37]. Thus, while reinfection with varicella is suspected to be infrequent, confirmation of this fact is difficult given errors in reporting and diagnosis.

Hence, while other sources of periodicity, including seasonally varying transmissibility may appear more likely explanations of varicella periodicity, outcomes from our model appear to accord with data on recurrence and reinfection, suggesting a role for these processes in enhanced models of VZV transmission.

We must acknowledge substantial limitations in relation to the realism exhibited by our model. It is generally accepted that age-dependent transmission is important in the context of varicella and HZ transmission and disease incidence but we do not include this here. Instead we include only a basic demographic process involving a constant birth/death rate *μ* as our approach aimed to isolate the impacts of recurrence and reinfection in a prototype model via minimising the total number of model parameters (a similar approach was used in, for example, [18]). A more realistic model would incorporate age-dependent transmission rates and age-dependent risks of HZ reactivation, with potential to consider more subtle differences such as gender related risks of disease. Other models also implement the assumption that breakthrough varicella is milder and less contagious than primary infection, based on experience with vaccine trials and implementation. Consideration of the impact of vaccination is also ignored here, given that the focus is on qualitative behaviours in a simple model. Note that as a result of the mentioned limitations, varicella incidence in the entire population produced by our model is higher than zoster incidence (see Figure 3). This is typical in the absence of vaccination, while in the post-vaccination period one often observes zoster incidence increasing and exceeding that of varicella (see, for example, [17,38]). The increase in zoster could be further facilitated by the ageing of population as discussed in [23].

Periodicity of varicella and HZ as such do not seem to be of major consequences to public health. This is partly because periodicity of zoster has yet to be firmly substantiated. For example, an Italian study found an annual cycle in HZ notifications with significant peaks in May-June [22]. On the other hand, HZ periodicity was not detected by a number of population-based studies and long-term analyses of data [24,25]. However, there has been a steady interest in gaining a better understanding of the reasons for VZV reactivation - should that be successful, practical implications are likely to follow. It is possible that seasonal variations in the incidence of immunologically related diseases may be related to circannual variations in immune response [39] or, in the case of HZ, to the effects of ultraviolet (UV) [40]. We suggest that varicella and zoster periodicity may be potentially explained solely by VZV natural history and including clinical reinfection with varicella and the possibility that only a limited exposure is needed for immune boosting in modelling studies may result in more accurate predictions.

## Additional file

Additional file 1 **Supplementary materials.** Hopf curves for all parameter pairs (78 figures).
