# Supplementary material for: Periodicity of varicella-zoster virus in the presence of immune boosting and clinical reinfection with varicella
Source: Theor Biol Med Model. 2015 Apr 11;12:6. doi: 10.1186/s12976-015-0002-5 (PMC4399247; doi:10.1186/s12976-015-0002-5)
Supplement: Supplementary file 1 — Supplementary materials. Hopf curves for all parameter pairs (78 figures). [file 12976_2015_2_MOESM1_ESM.pdf]

Periodicity of varicella-zoster virus in the presence of immune boosting and clinical reinfection with varicella

Supplementary Materials

Hopf curves for all parameter pairs (78 figures)

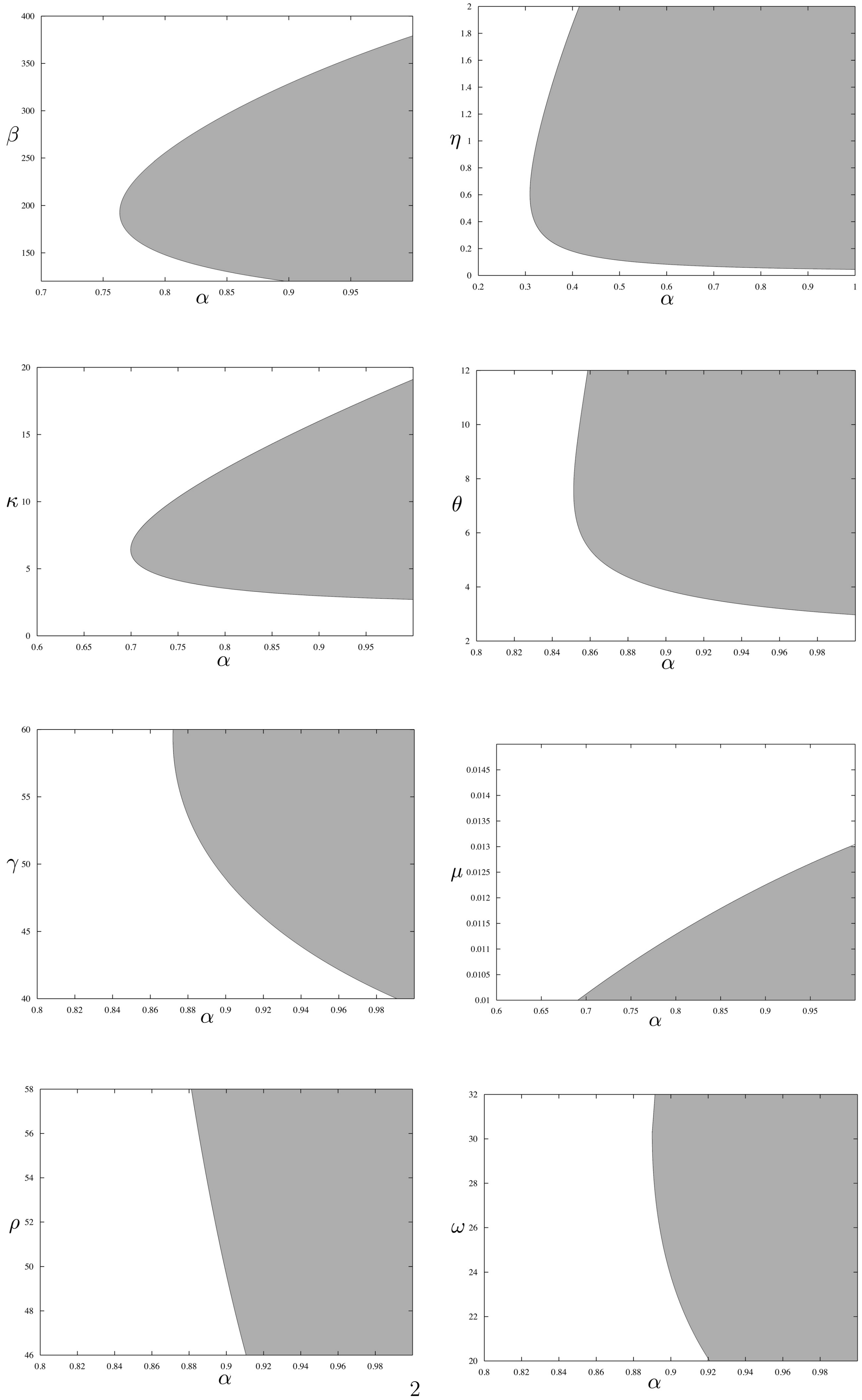
β
α
η
α
κ
α
θ
α
γ
α
μ
α
ρ
α
ω
α

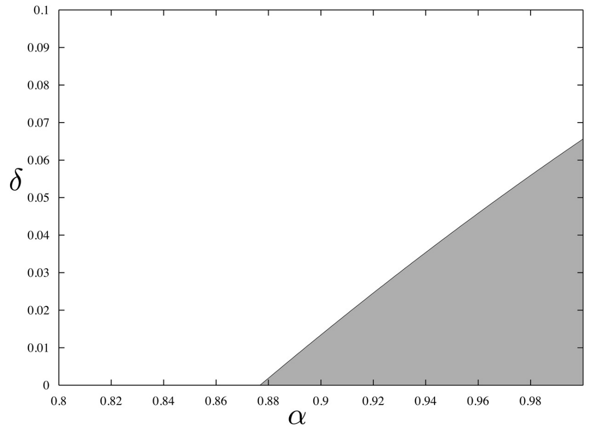
δ
α
0 0.01 0.02 0.03 0.04 0.05 0.06 0.07 0.08 0.09 0.1
0.8 0.82 0.84 0.86 0.88 0.9 0.92 0.94 0.96 0.98

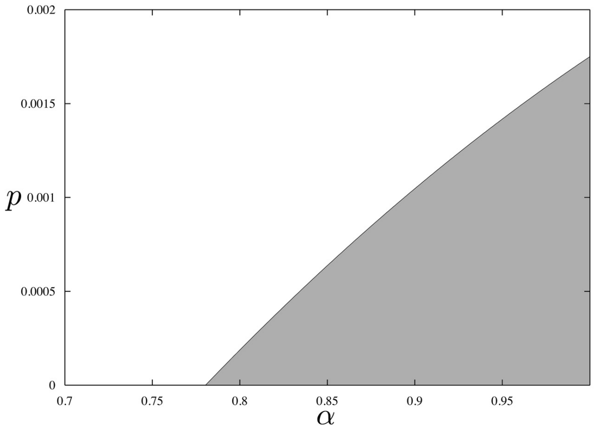
p
α
0 0.0005 0.001 0.0015 0.002
0.7 0.75 0.8 0.85 0.9 0.95

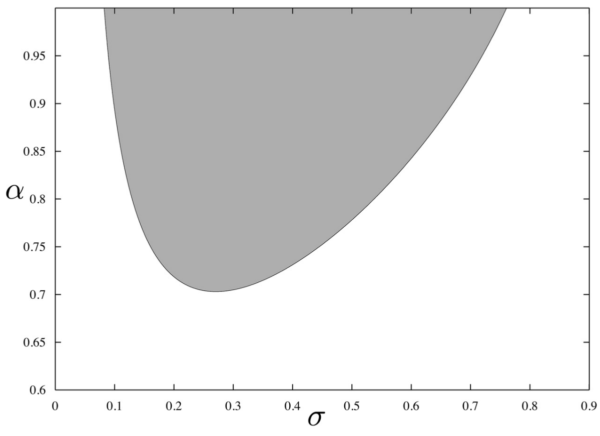
α
σ
0.6 0.65 0.7 0.75 0.8 0.85 0.9 0.95
0 0.1 0.2 0.3 0.4 0.5 0.6 0.7 0.8 0.9

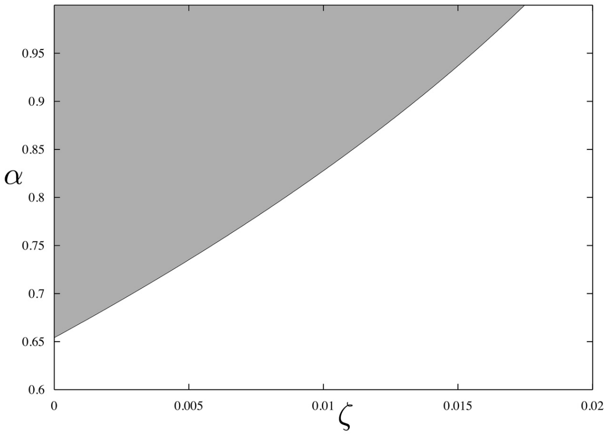
α
ζ
0.6 0.65 0.7 0.75 0.8 0.85 0.9 0.95
0 0.005 0.01 0.015 0.02

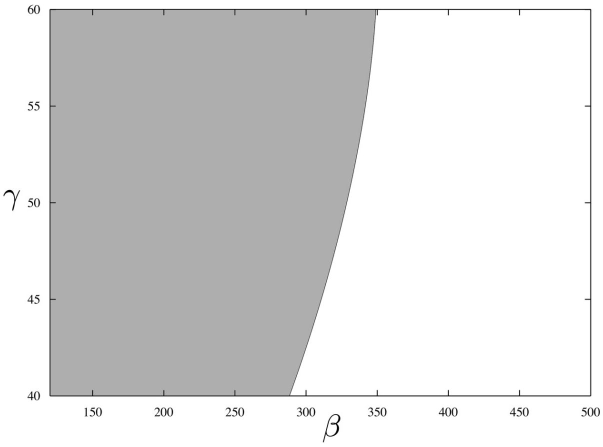
γ
β
40 45 50 55 60
150 200 250 300 350 400 450 500

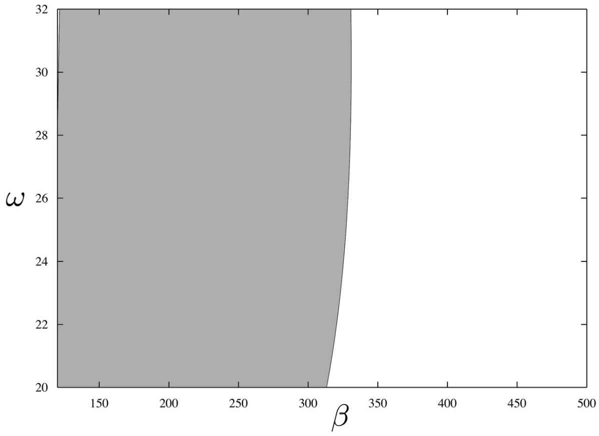
ω
β
20 22 24 26 28 30 32
150 200 250 300 350 400 450 500

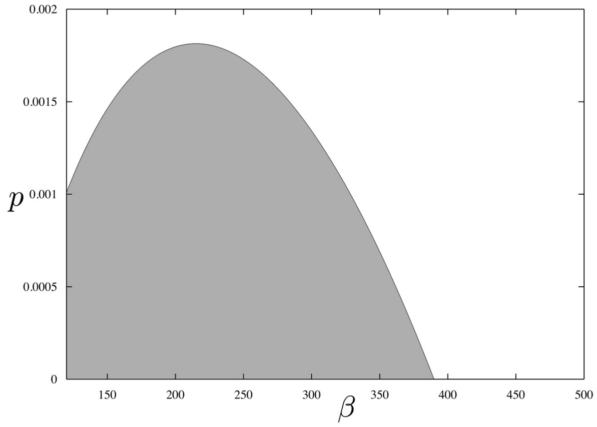
p
β
0 0.0005 0.001 0.0015 0.002
150 200 250 300 350 400 450 500

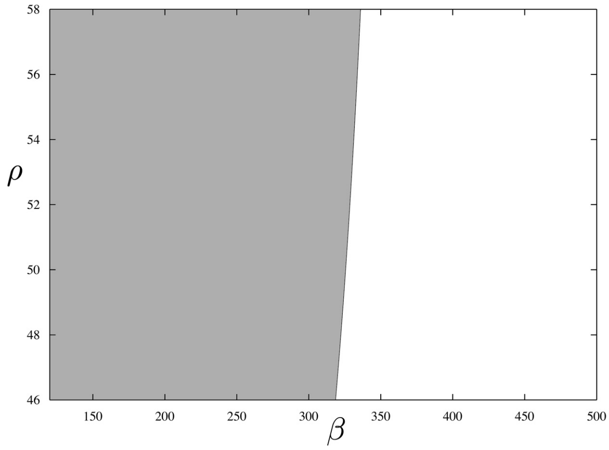
ρ
β
46 48 50 52 54 56 58
150 200 250 300 350 400 450 500

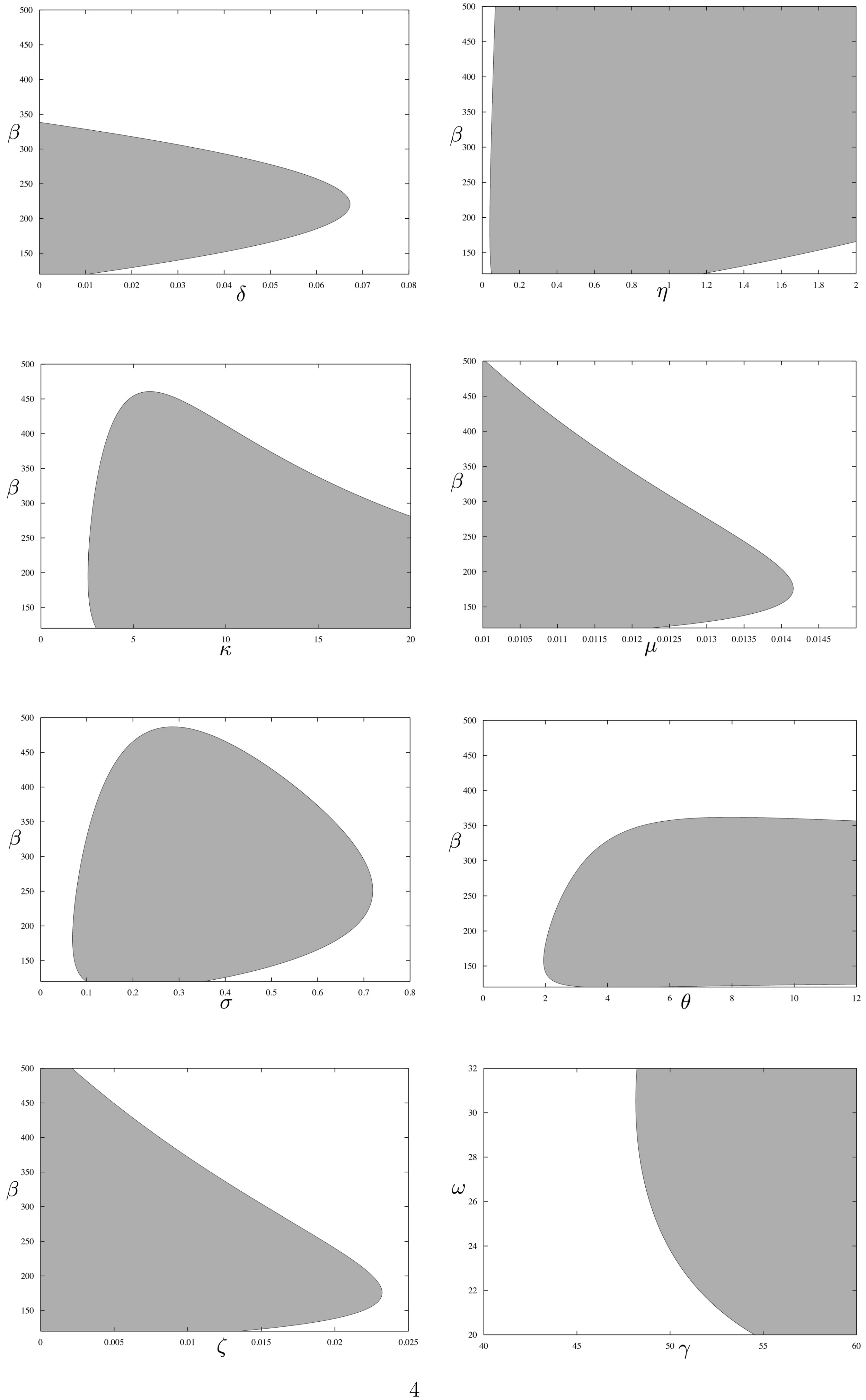
β
δ
0 0.01 0.02 0.03 0.04 0.05 0.06 0.07 0.08
150 200 250 300 350 400 450 500
β
η
0 0.2 0.4 0.6 0.8 1 1.2 1.4 1.6 1.8 2
β
κ
0 5 10 15 20
β
μ
0.01 0.0105 0.011 0.0115 0.012 0.0125 0.013 0.0135 0.014 0.0145
β
σ
0 0.1 0.2 0.3 0.4 0.5 0.6 0.7 0.8
β
θ
0 2 4 6 8 10 12
β
ζ
0 0.005 0.01 0.015 0.02 0.025
ω
γ
40 45 50 55 60
20 22 24 26 28 30 32

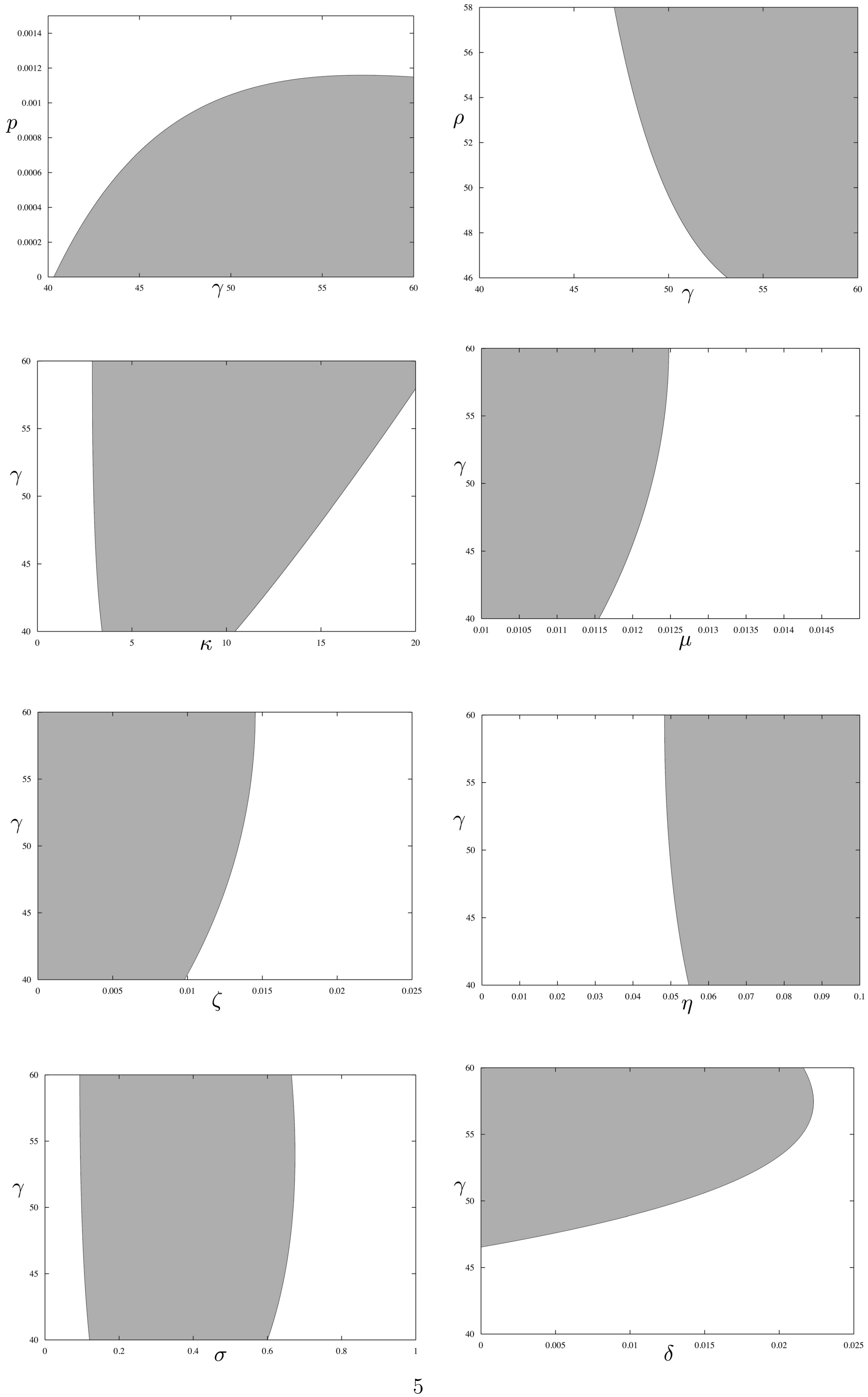
p
γ
ρ
γ
γ
κ
γ
μ
γ
ζ
γ
η
γ
σ
γ
δ

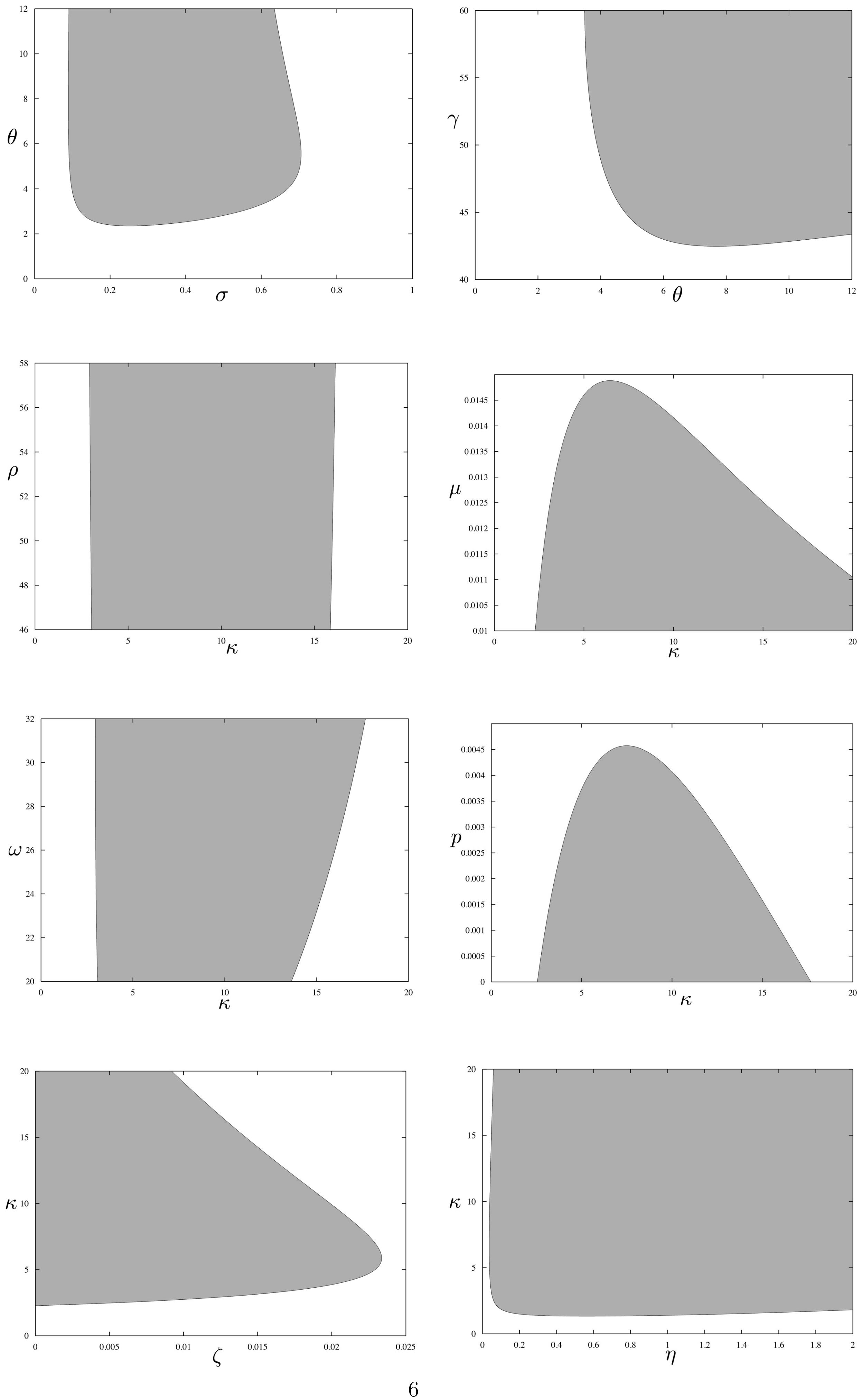
θ
0 2 4 6 8 10 12
σ
0 0.2 0.4 0.6 0.8 1
γ
40 45 50 55 60
θ
0 2 4 6 8 10 12
ρ
46 48 50 52 54 56 58
κ
0 5 10 15 20
μ
0.01 0.0105 0.011 0.0115 0.012 0.0125 0.013 0.0135 0.014 0.0145
κ
0 5 10 15 20
ω
20 22 24 26 28 30 32
κ
0 5 10 15 20
p
0 0.0005 0.001 0.0015 0.002 0.0025 0.003 0.0035 0.004 0.0045
κ
0 5 10 15 20
κ
0 5 10 15 20
ζ
0 0.005 0.01 0.015 0.02 0.025
κ
0 5 10 15 20
η
0 0.2 0.4 0.6 0.8 1 1.2 1.4 1.6 1.8 2

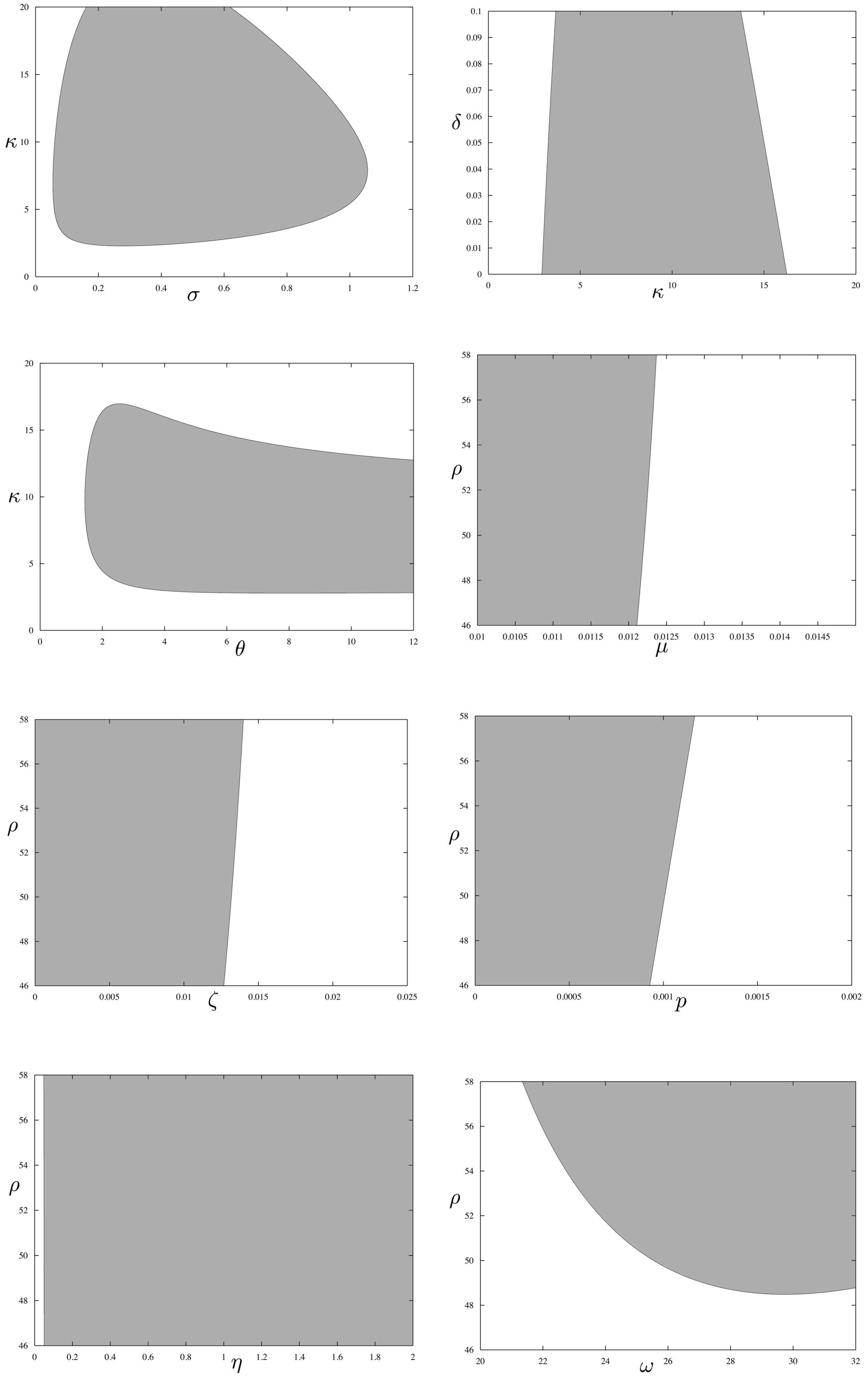
κ
σ
δ
κ
κ
θ
ρ
μ
ρ
ζ
ρ
p
ρ
η
ρ
ω

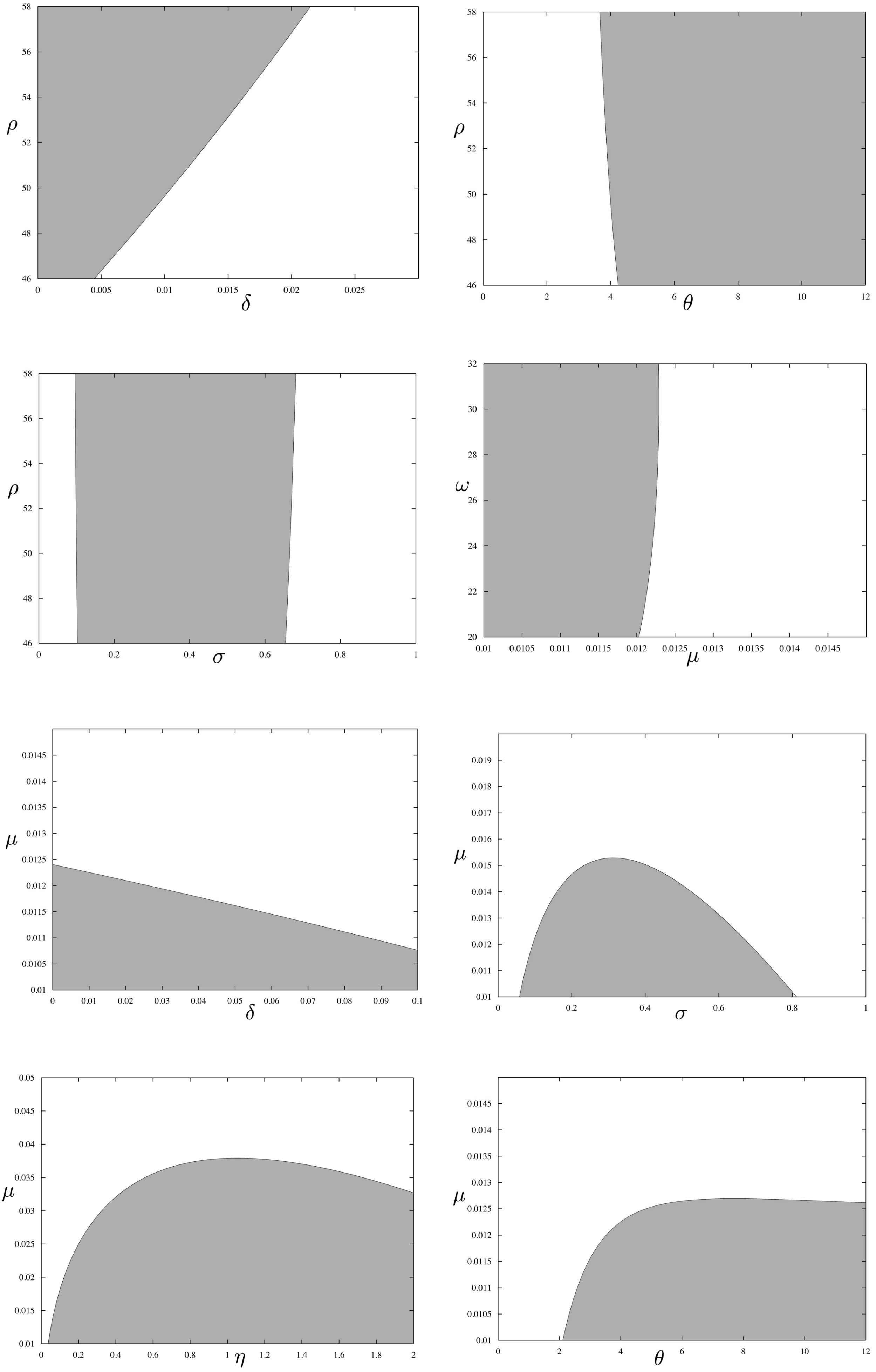
ρ
58 56 54 52 50 48 46
0 0.005 0.01 0.015 0.02 0.025
δ
ρ
58 56 54 52 50 48 46
0 2 4 6 8 10 12
θ
ρ
58 56 54 52 50 48 46
0 0.2 0.4 0.6 0.8 1
σ
ω
32 30 28 26 24 22 20
0.01 0.0105 0.011 0.0115 0.012 0.0125 0.013 0.0135 0.014 0.0145
μ
μ
0.0145 0.014 0.0135 0.013 0.0125 0.012 0.0115 0.011 0.0105 0.01
0 0.01 0.02 0.03 0.04 0.05 0.06 0.07 0.08 0.09 0.1
δ
μ
0.019 0.018 0.017 0.016 0.015 0.014 0.013 0.012 0.011 0.01
0 0.2 0.4 0.6 0.8 1
σ
μ
0.05 0.045 0.04 0.035 0.03 0.025 0.02 0.015 0.01
0 0.2 0.4 0.6 0.8 1 1.2 1.4 1.6 1.8 2
η
μ
0.0145 0.014 0.0135 0.013 0.0125 0.012 0.0115 0.011 0.0105 0.01
0 2 4 6 8 10 12
θ

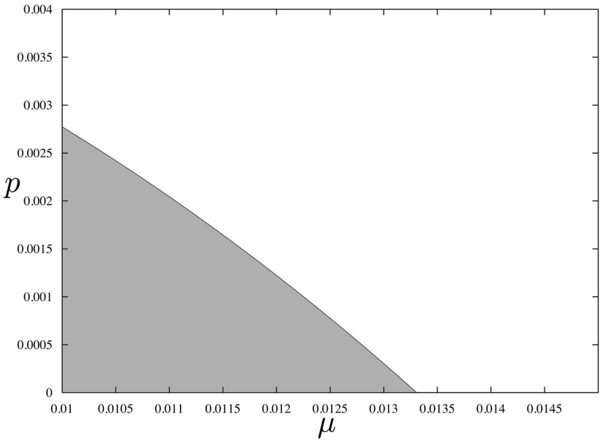
p
μ
0.004 0.0035 0.003 0.0025 0.002 0.0015 0.001 0.0005 0
0.01 0.0105 0.011 0.0115 0.012 0.0125 0.013 0.0135 0.014 0.0145

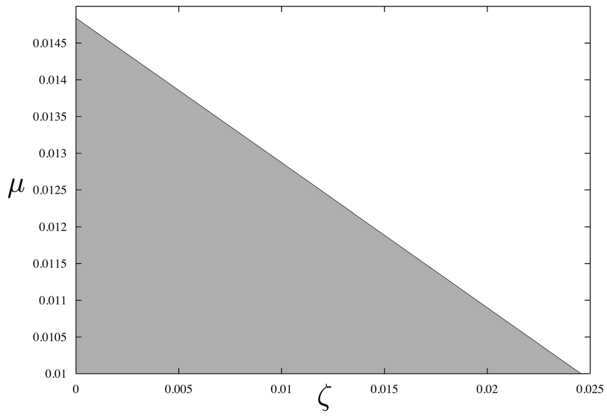
μ
ζ
0.0145 0.014 0.0135 0.013 0.0125 0.012 0.0115 0.011 0.0105 0.01
0 0.005 0.01 0.015 0.02 0.025

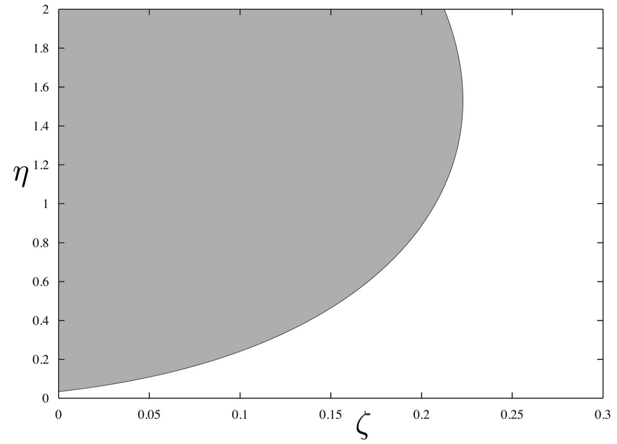
η
ζ
2 1.8 1.6 1.4 1.2 1 0.8 0.6 0.4 0.2 0
0 0.05 0.1 0.15 0.2 0.25 0.3

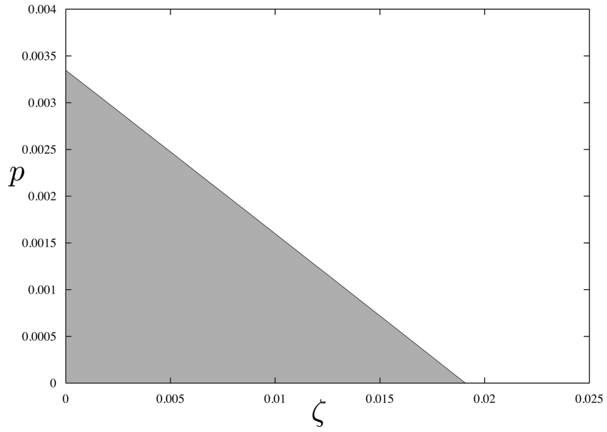
p
ζ
0.004 0.0035 0.003 0.0025 0.002 0.0015 0.001 0.0005 0
0 0.005 0.01 0.015 0.02 0.025

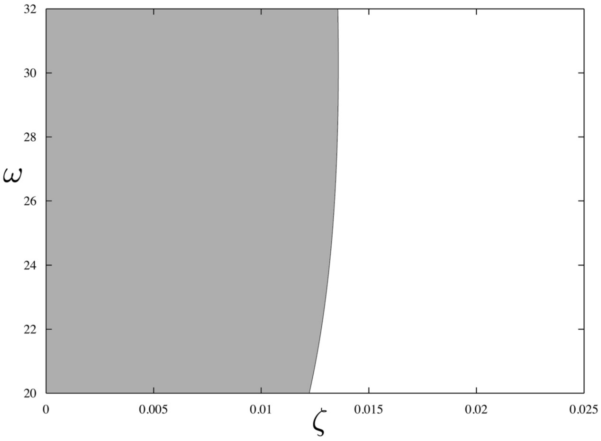
ω
ζ
32 30 28 26 24 22 20
0 0.005 0.01 0.015 0.02 0.025

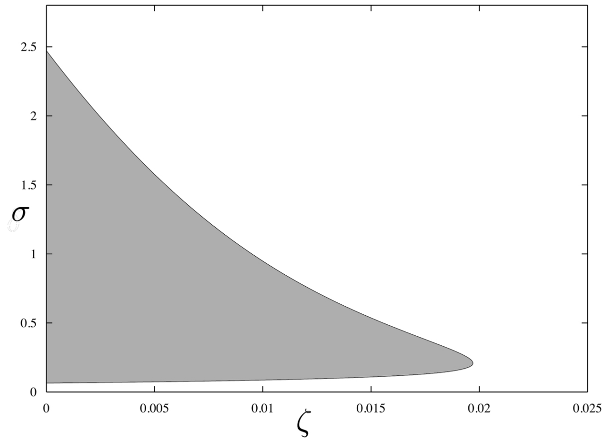
σ
ζ
2.5 2 1.5 1 0.5 0
0 0.005 0.01 0.015 0.02 0.025

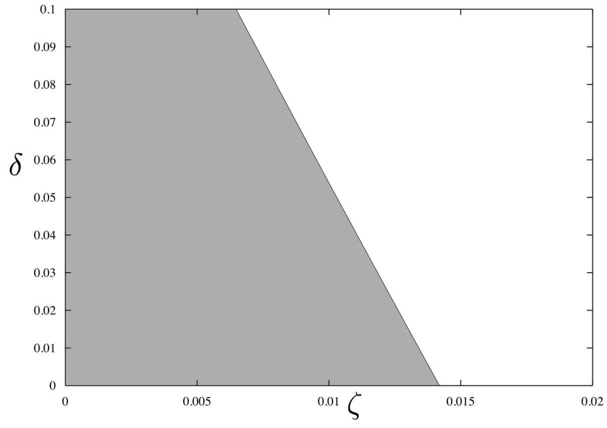
δ
ζ
0.1 0.09 0.08 0.07 0.06 0.05 0.04 0.03 0.02 0.01 0
0 0.005 0.01 0.015 0.02

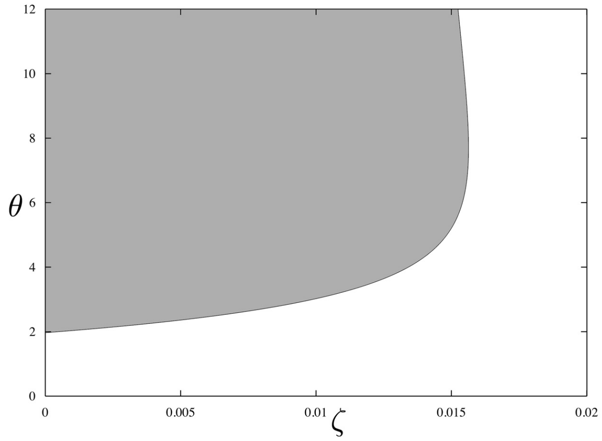
θ
ζ
12 10 8 6 4 2 0
0 0.005 0.01 0.015 0.02

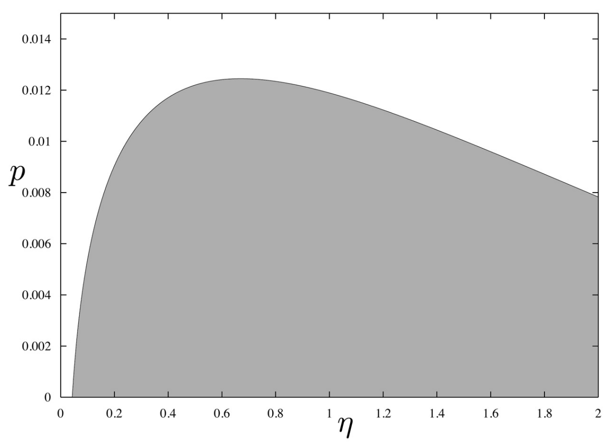
p
η
0
0.002
0.004
0.006
0.008
0.01
0.012
0.014
0.2
0.4
0.6
0.8
1
1.2
1.4
1.6
1.8
2

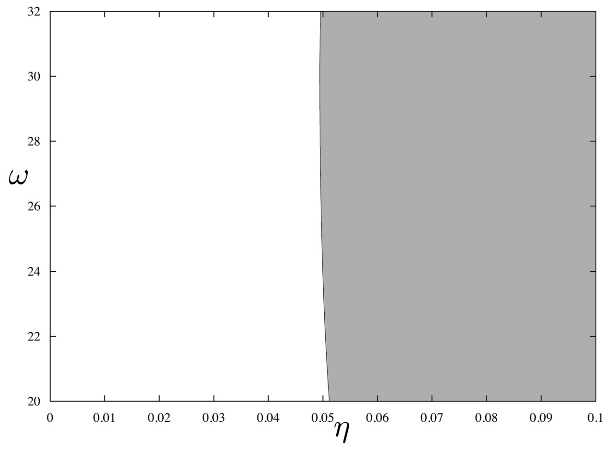
ω
η
20
22
24
26
28
30
32
0
0.01
0.02
0.03
0.04
0.05
0.06
0.07
0.08
0.09
0.1

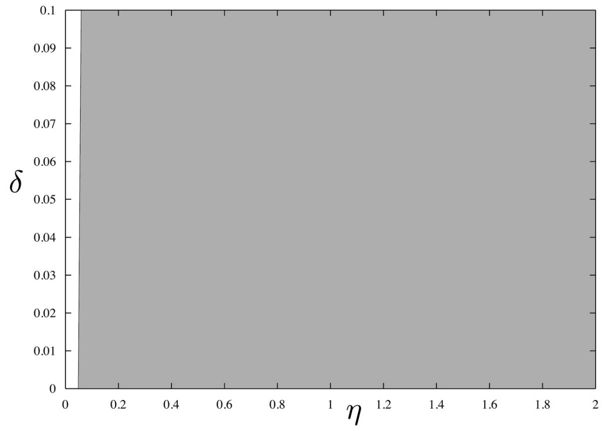
δ
η
0
0.01
0.02
0.03
0.04
0.05
0.06
0.07
0.08
0.09
0.1
0.2
0.4
0.6
0.8
1
1.2
1.4
1.6
1.8
2

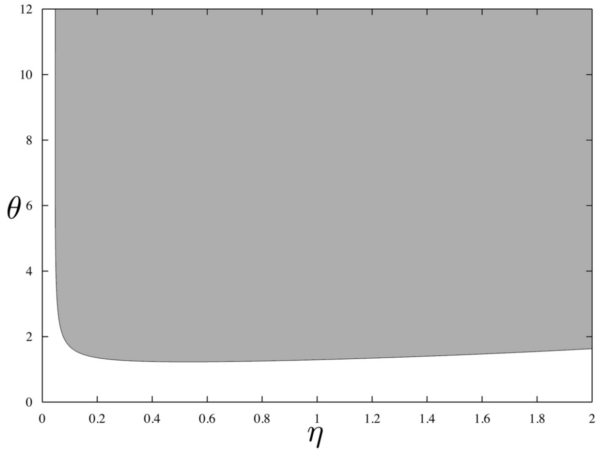
θ
η
0
2
4
6
8
10
12
0.2
0.4
0.6
0.8
1
1.2
1.4
1.6
1.8
2

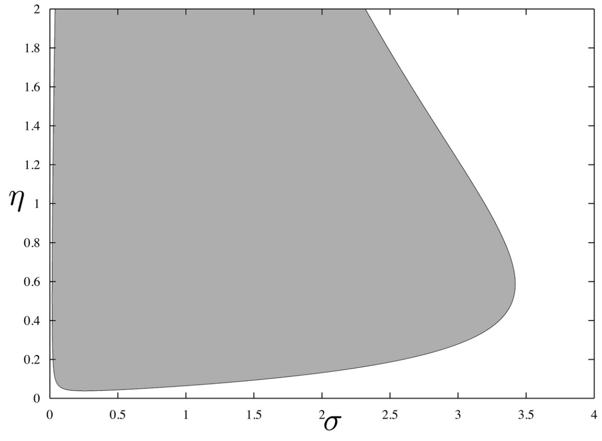
η
σ
0
0.2
0.4
0.6
0.8
1
1.2
1.4
1.6
1.8
2
0.5
1.5
2.5
3
3.5
4

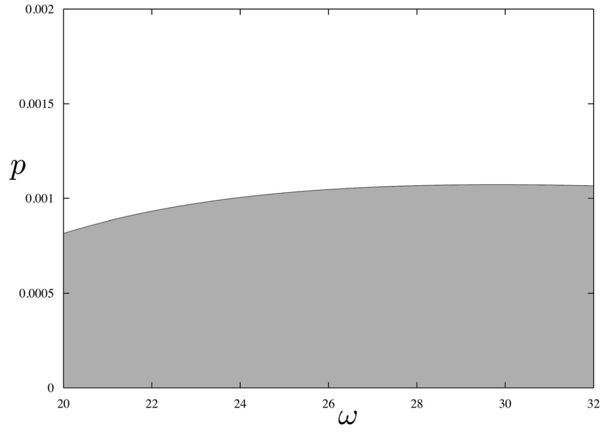
p
ω
0
0.0005
0.001
0.0015
0.002
20
22
24
26
28
30
32

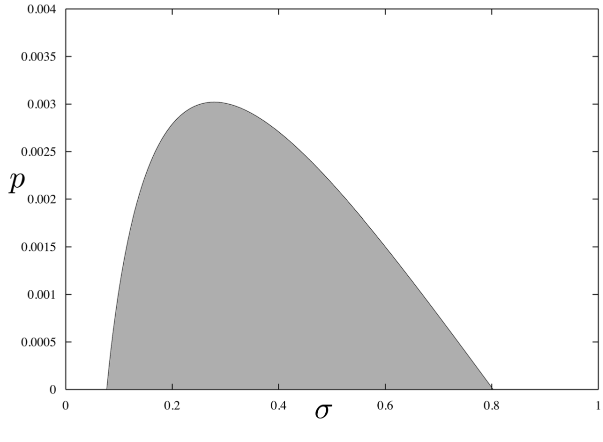
p
σ
0
0.0005
0.001
0.0015
0.002
0.0025
0.003
0.0035
0.004
0.2
0.4
0.6
0.8
1

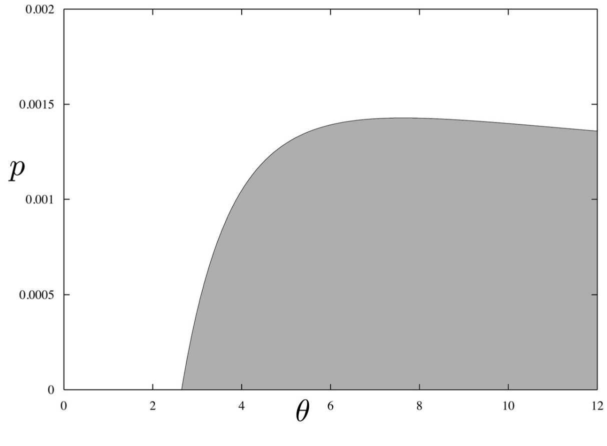
p
θ
0
0.0005
0.001
0.0015
0.002
2
4
6
8
10
12

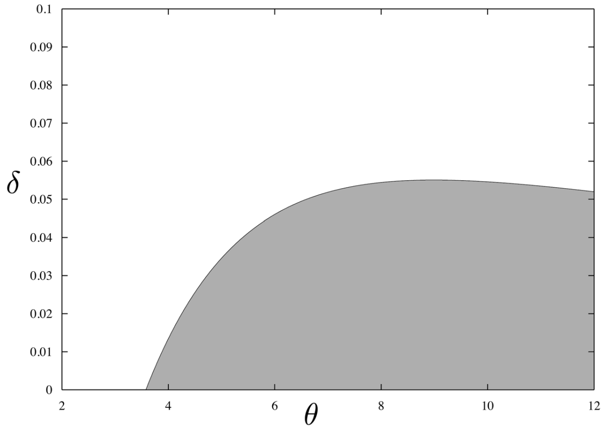
δ
θ
0.1
0.09
0.08
0.07
0.06
0.05
0.04
0.03
0.02
0.01
0
2
4
6
8
10
12

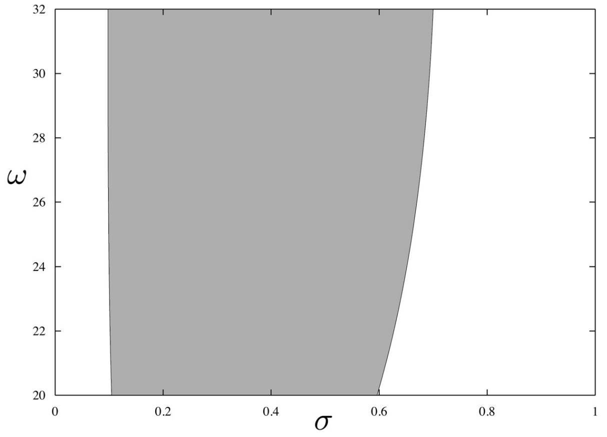
ω
σ
32
30
28
26
24
22
20
0
0.2
0.4
0.6
0.8
1

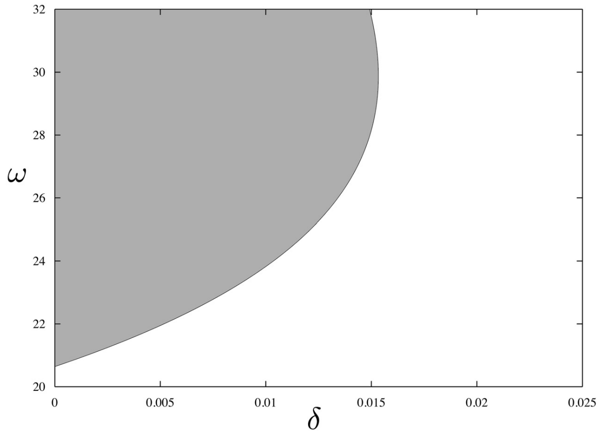
ω
δ
32
30
28
26
24
22
20
0
0.005
0.01
0.015
0.02
0.025

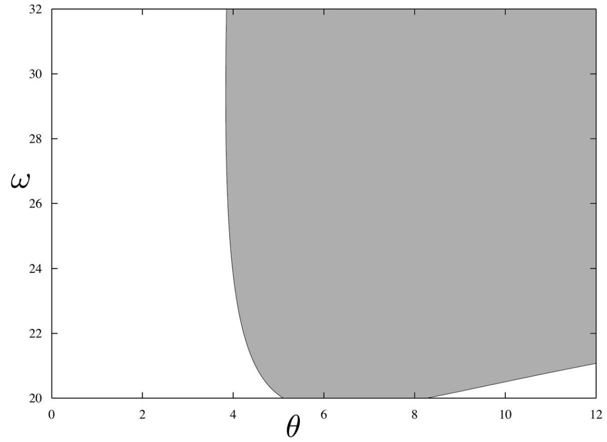
ω
θ
32
30
28
26
24
22
20
0
2
4
6
8
10
12

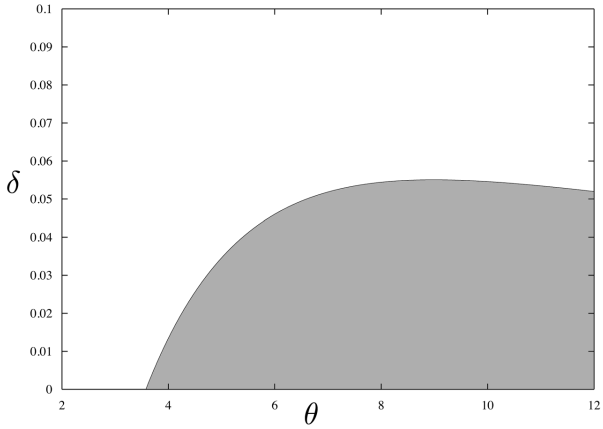
δ
θ
0.1
0.09
0.08
0.07
0.06
0.05
0.04
0.03
0.02
0.01
0
2
4
6
8
10
12

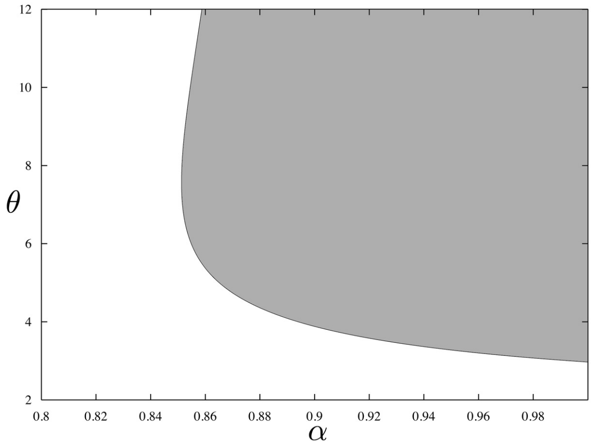
θ
α
12
10
8
6
4
2
0.8
0.82
0.84
0.86
0.88
0.9
0.92
0.94
0.96
0.98
